# Airway Management in Fontan Patients with LPA Stenosis

**DOI:** 10.3390/jcdd13090463

**Published:** 2026-09-14

**Authors:** Emma Mavinkurve, Gregor Krings, Mirella Molenschot, Linda van Wagenberg, Johannes Breur

**Affiliations:** 1 Utrecht University, 3584 CS Utrecht, The Netherlands; e.j.t.mavinkurve@students.uu.nl; 2 Pediatric Cardiology, Wilhelmina Children’s Hospital, Universuty Medical Center, 3508 AB Utrecht, The Netherlands; g.krings@umcutrecht.nl (G.K.); m.m.c.molenschot@umcutrecht.nl (M.M.); l.vanwagenberg@umcutrecht.nl (L.v.W.)

**Keywords:** congenital heart disease, 3DRA, 3 dimensional rotational angiography, percutaneous interventions, single ventricle

## Abstract

Left pulmonary artery (LPA) stenosis is encountered in up to 65% of patients with a Fontan circulation. Stenting of LPA stenosis can be complicated by compression of the left bronchus. We evaluated the effect of periprocedural airway management with three-dimensional rotational angiography (3DRA) and bronchoscopy on the occurrence of left main bronchus compression in Fontan patients undergoing percutaneous treatment for LPA stenosis. All pediatric cardiac catheterizations for LPA stenosis between 2011 and 2024 as well as the pre- and postoperative course were analyzed. In 64 of a total of 109 procedures airway imaging was used (3DRA (*n* = 50), bronchoscopy (*n* = 1), bronchoscopy and 3DRA (*n* = 13)). Airway imaging led to an adaptation of the intervention in 43 cases; stent ovalization was performed in 23 cases, balloon interrogation with simultaneous airway visualization was performed in 24 cases and the intended procedure was abandoned in two cases. In six cases balloon dilatation of a compressed left main bronchus was performed with mild success in two cases where balloon dilatation led to altered LPA stent morphology increasing the lumen of the left main bronchus. Thus, the left main bronchus is at high risk for compression when LPA stenting is performed in patients with a Fontan circulation. Periprocedural imaging of the airway has the potential to prevent airway compression. Bronchus dilatation offered no successful treatment for airway compression by an LPA stent.

## 1. Introduction

Patients with single ventricle (SV) physiology are palliated with the Fontan or Total Cavo Pulmonary Connection (TCPC) procedure. In patients with a Fontan circulation, unobstructed pulmonary blood flow is crucial for maintaining low pulmonary vascular resistance and optimal Fontan hemodynamics [1]. However, left pulmonary artery (LPA) stenosis is a common complication seen in 35–65% of patients often treated with LPA stenting [2,3,4]. The left main bronchus (LMB) is located between the neo-aortic root, the left pulmonary artery and the descending aorta. Stenting the LPA in this relatively fixed space may lead to LMB compression [5]. Previous studies using three-dimensional rotational angiography (3DRA) have demonstrated that LMB compression can already be present in patients with SV physiology and LPA stenosis, even before stent implantation. The Damus–Kaye–Stansel (DKS) operation is especially associated with pre-existing bronchial compression [6,7]. This is extremely important when considering LPA stenting in SV patients depending on low pulmonary vascular resistance and normal lung function for optimal long-term outcome. Conventional methods for airway management during cardiac catheterization are bronchoscopy and two-dimensional angiography. In patients with an increased risk of airway compression pre-procedural bronchoscopy is used to view the LMB during balloon interrogation of the LPA. When the airway remains patent, stent angioplasty can be performed [5]. A more recent technique is 3DRA which can be used to visualize the anatomical relationship between vascular structures and airway anatomy during cardiac catheterization. This creates a multiplanar reconstruction (MPR) making it possible to view the anatomy in any orientation (axial, sagittal or coronal) allowing determination of the exact point of caliber change in airway [8]. This more advanced imaging may lead to an adaption of the intervention, such as ovalization of the LPA stent. This novel technique, first described by Krings et al., proved successful in treating LPA stenosis in patients at risk of bronchial compression, guaranteeing patency of the LMB [7].

Detailed assessment of the vessel–vessel and vessel–airway relationship may be crucial during treatment of LPA stenosis. This study aims to investigate the periprocedural and long-term consequences of airway imaging in the treatment of LPA stenosis in Fontan patients.

## 2. Materials and Methods

### 2.1. Study Design

In this retrospective cohort study, all SV patients undergoing percutaneous procedures with intended LPA dilatation or stenting at the Wilhelmina Children’s Hospital (WKZ)/University Medical Center Utrecht, Utrecht, the Netherlands, from 2011 to 2024 were consecutively enrolled. In patients with reported LMB compression follow-up was continued until 2025. All procedures were reviewed systematically and follow-up data was extracted from patient files. The primary outcome of this study was the occurrence of intervention adaptation in cases where airway imaging was performed. The secondary outcome is the occurrence of airway compression-related symptoms.

### 2.2. Procedure Description and Image Acquisition

**Figure 1:**
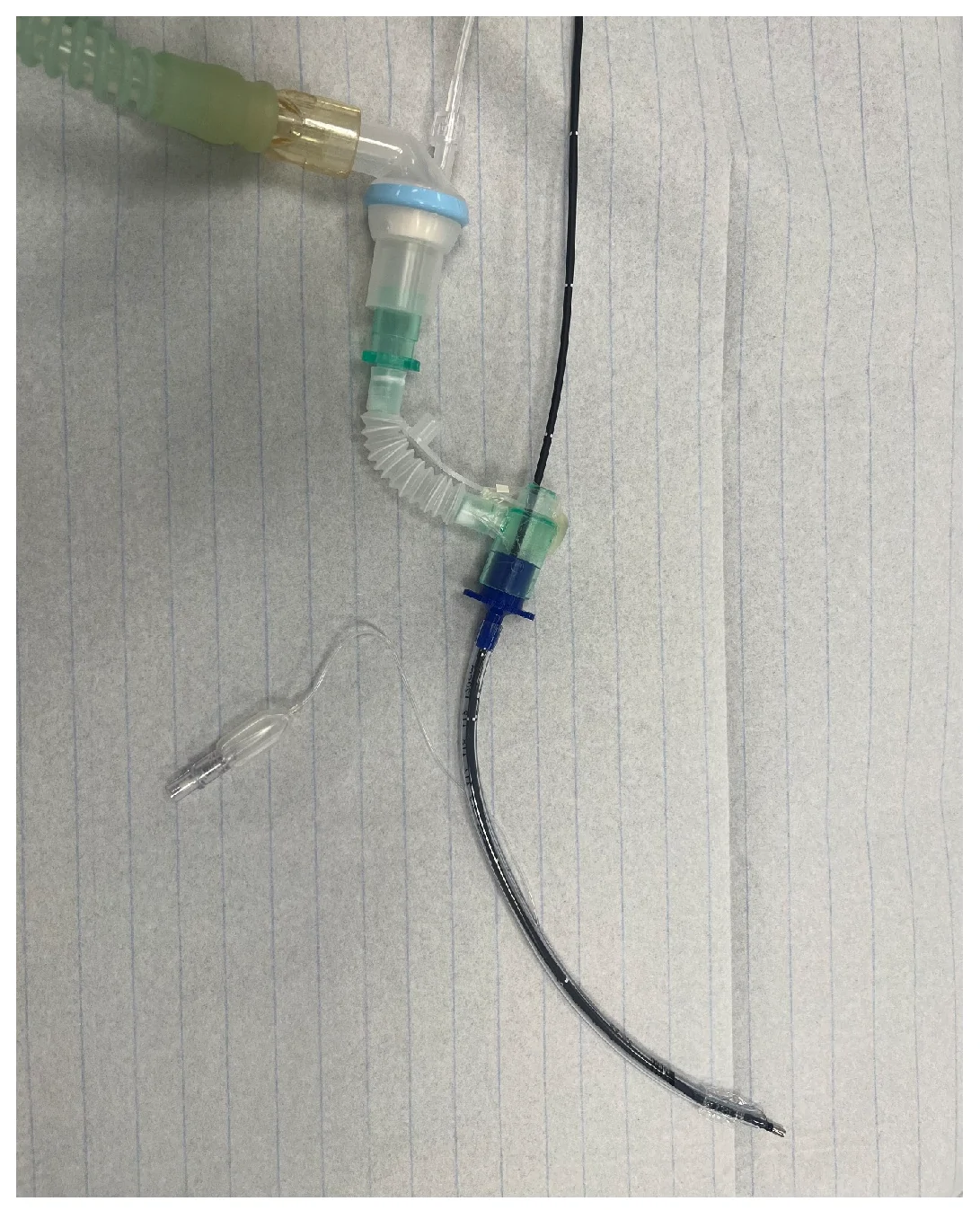
Bronchoscopy setup. A flexible intubation video endoscope is inserted through the tube to visualize the LMB.

Cardiac catheterization was performed under general anesthesia in all patients. Complete hemodynamic assessment was performed, including pulmonary wedge pressures and pressure pullback loops. 3DRA was obtained using the Artis zee system with syngo DynaCT (Siemens Healthineers, Forchheim, Germany) (2011–2023) or Artis Q.zen (Siemens Healthineers, Forchheim, Germany) (2023 and onwards) and our 3DRA univentricular heart protocol [9]. Post-processing routinely included heart, vessel–vessel and vessel–airway interactions. In addition, in the retro-aortic space, the distance between the LPA and neo-aorta, coronary arteries, LMB and descending aorta was assessed in anteroposterior, lateral and cranio-caudal orientation. If necessary, an additional “empty” 3DRA (3DRA without contrast solely to visualize airway, other stents in situ and contrast-filled balloon) was made in unstented patients using a single balloon with the target stent diameter in the LPA to evaluate future airway patency. After these diagnostics the interventional procedure was performed. Airway patency was evaluated post stent ovalization by “empty” 3DRA or bronchoscopy. Pressure pullbacks were performed to assess residual gradients. Bronchoscopy was performed by the pediatric cardiac anesthesiologist using a flexible intubation video endoscope (Karl Storz 3.0 or 4.0 mm outer diameter, Karl Storz SE & Co, Tuttlingen, Germany) inserted through the tube to visualize the LMB (Figure 1).

### 2.3. Data Collection

Patient characteristics were extracted from Filemaker Pro, including age, height, type of stent and implantation balloon, diagnosis, type of surgery, invasive blood pressures (vena cava superior (VCS), vena cava inferior (VCI), LPA, right pulmonary artery (RPA), end-diastolic pressure (EDp), Wedge, gradient), airway imaging (bronchoscopy/3DRA/nothing), airway intervention, outcome (gradient reduction, morphologically, complications, re-intervention and radiation data).

Baseline characteristics represent the first catheterization performed. Descriptive statistics were used to present the data and median and range were calculated. There was no correction for missing data. Adaption of intervention was defined by either balloon interrogation, ovalization, no stent/dilatation or a combination of both due to proximity of the LMB. The severity of stenosis was categorized as mild (less than 25%), moderate (25–75%) and severe (more than 75%) compared with the diameter of the adjacent normal airway [10]. Clinical outcome on long-term follow-up was extracted from the patient files and was defined as airway symptoms caused by the placement of the LPA stent, which directly led to bronchus compression. Airway symptoms classified as antibiotic prophylaxis, recurrent respiratory infections, other airway medication such as nebulizers, clinical symptoms (e.g., dyspnea) and other pulmonary imaging after the intervention (e.g., lung function, exercise test, CT, VP-scan).

## 3. Results

### 3.1. Airway Management

A total of 109 procedures from 68 patients were included. Characteristics of this study population are presented in Table 1.

Table 2 presents periprocedural Fontan hemodynamics (Table 2).

In all but one patient, stenting of the LPA was performed during the first catheterization. The EV3 MegaLD stent and the Cook Formula 535 stent were used in the majority of the patients (Table 3). In one patient LPA stenting was refrained from due to complete compression of the LMB, during balloon interrogation visible on 3DRA. Balloon interrogation was performed with Advance (*n* = 14), Powerflex (n = 5), BiB (*n* = 2) and CBV Bald (*n* = 1) balloons.

**Figure 2:**
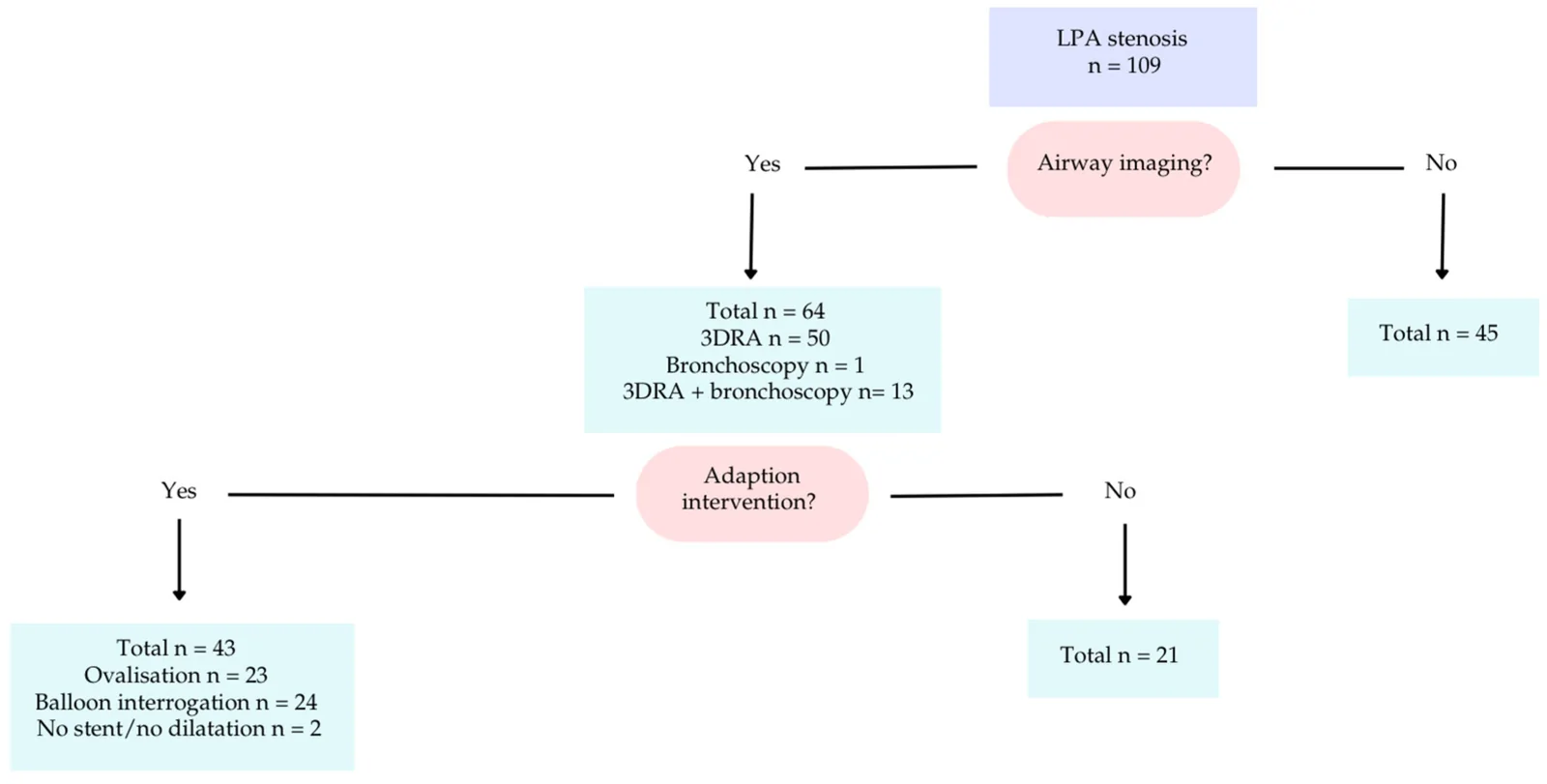
Flowchart influence airway management on adaption of intervention.

**Figure 3:**
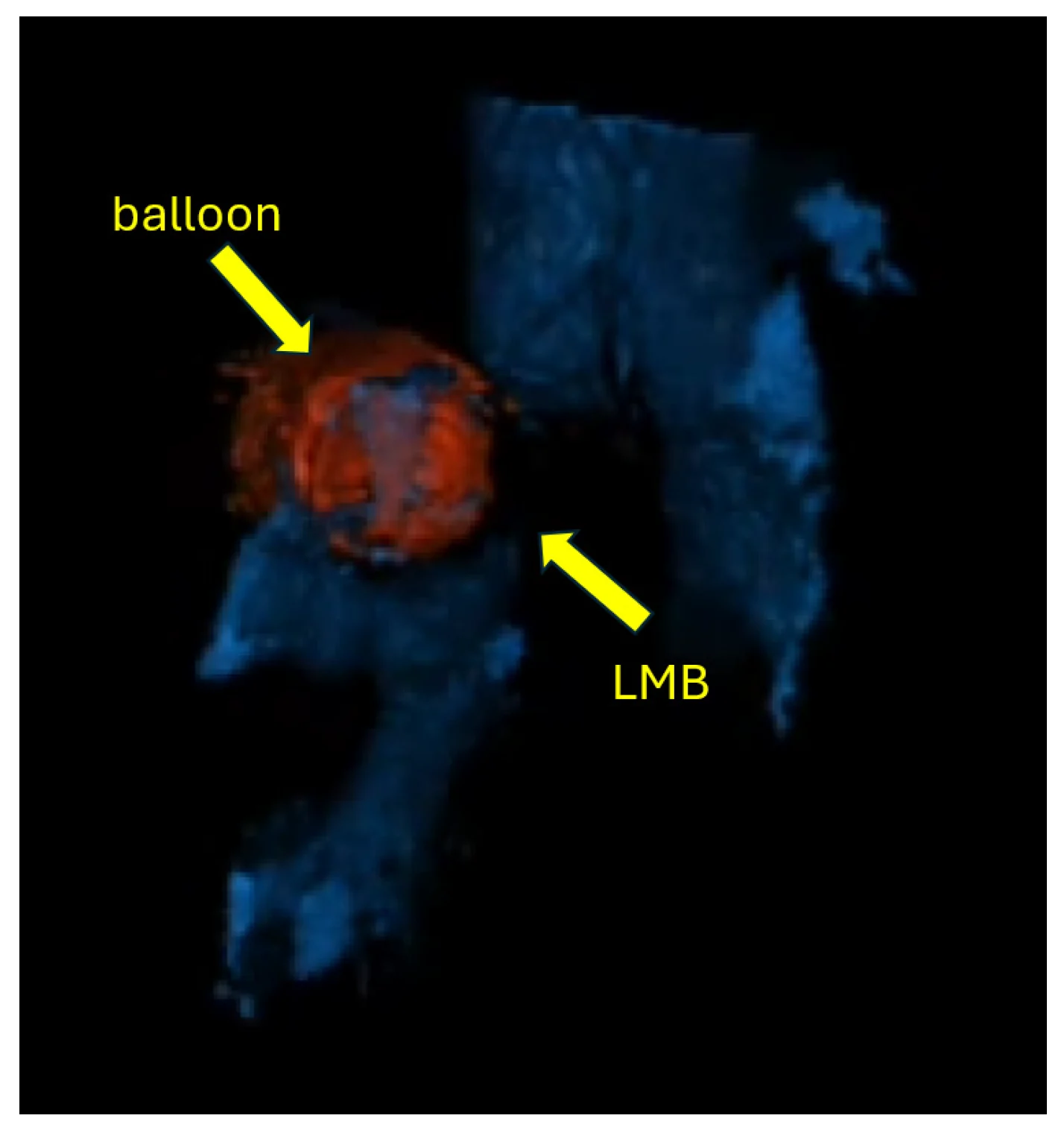
Lateral 3DRA view of a contrast-filled balloon in the LPA (orange) and almost complete compression of the LMB (blue) during LPA balloon interrogation.

**Figure 4:**
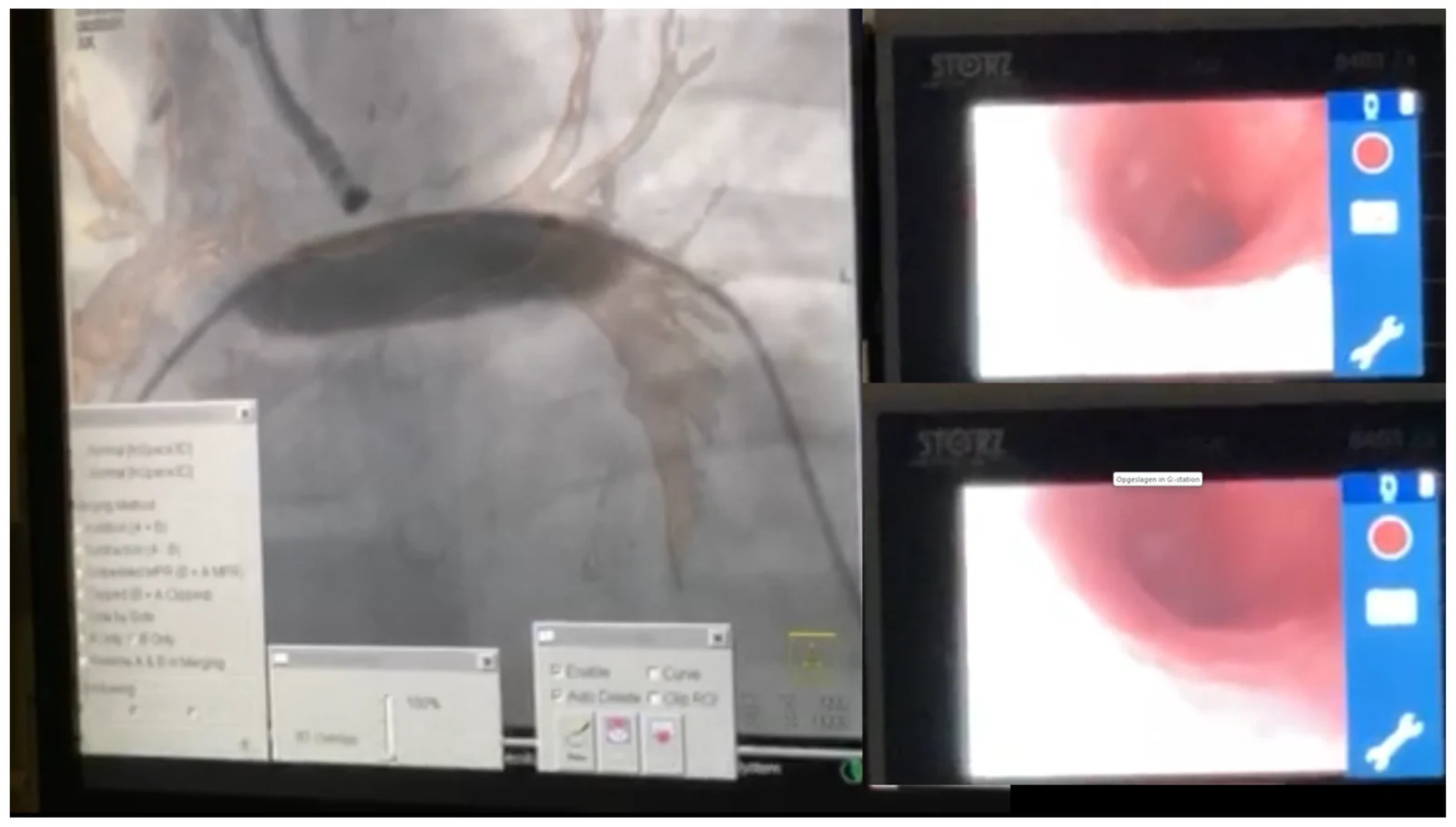
Periprocedural anterior–posterior (AP) image of 3DRA roadmap during balloon interrogation (**left**) and bronchoscopy images before (**right upper**) and during balloon interrogation (**right lower**). Note the airway compression during balloon interrogation.

During 109 procedures airway imaging was reported in 64 procedures (Figure 2) ((3DRA *n* = 50 (Figure 3), bronchoscopy *n* = 1 and 3DRA + bronchoscopy *n* = 13 (Figure 4)). In 43 (67%) cases this led to an adaption of intervention (ovalization/balloon interrogation/no stent/no dilatation).

In this study 13 procedures used both 3DRA and bronchoscopy for airway imaging. 3DRA and bronchoscopy led to no difference in airway judgment in 10 cases, whereas a difference between both modalities was reported in three procedures, where 3DRA led to misleading information during balloon interrogation. This was caused by scattering and artifacts on 3DRA, caused by the contrast-filled balloon located directly next to the LMB.

### 3.2. Airway Compression

In total, 15 of 64 patients had some degree of LMB compression. In 14 patients LMB compression was present during the first procedure where airway imaging was performed. A previously placed stent was the cause of LMB compression in eight patients, a large DKS anastomosis in six patients. In one patient LMB compression was caused by stent placement. In this patient balloon interrogation with a 10 mm Advance balloon showed LMB compression. Therefore, direct oval stent implantation was performed using a 26 mm EV3 Mega LD stent on 2 8 × 20 mm Advance balloons. Bronchoscopy and 3DRA showed mild LMB compression after stent implantation. Currently this patient is symptom free and doing well.

### 3.3. Airway Interventions

**Figure 5:**
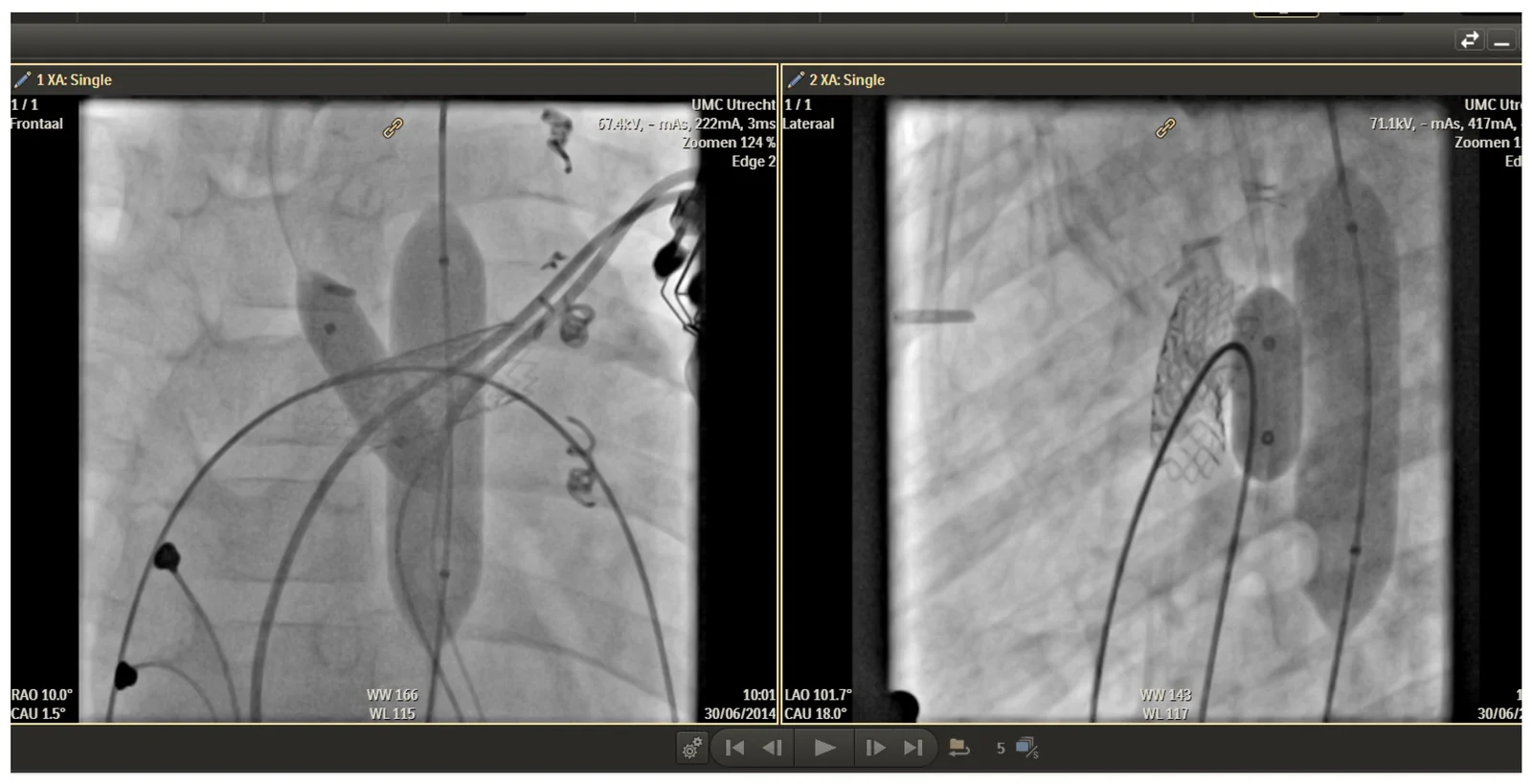
Anterior–posterior (AP) view (**left**) and lateral (**right**) image of simultaneous inflation of balloons in the left main bronchus (small balloon) and descending aorta (large balloon) to modulate the LPA stent. Also note the epicardial pacemaker wire and PM device in the AP view.

In four patients a total of seven LMB dilatations were performed (two patients one dilatation, one patient two dilatations, one patient three dilatations). The patient with three LMB dilatations presented at the age of 7 with severe compression of both the LPA and LMB due to a huge DKS anastomosis. After balloon interrogation with 8 mm (open airway) and 12 mm (occluded airway) balloons the LPA was stented with a 26 mm EV3 Mega LD stent on a 10 × 30 mm balloon. This resulted in occlusion of the LMB. LMB dilatation with a 6 × 20 mm Advance balloon was performed without success. This patient developed respiratory complaints and abnormal lung function tests. Therefore, a second procedure was performed with LMB dilatation with 8 and 10 mm balloons with simultaneous dilatation of the descending aorta to induce dorsal compression of the LPA stent. (Figure 5). Unfortunately, this had no effect on LMB patency.

Four years later the LPA stent was ovalized and LMB dilatation (10 mm advance balloon) with simultaneous manual sternal compression was performed. Again, this had no effect on airway patency. At latest follow-up, this patient is still using nebulizers to reduce symptoms.

In two patients LMB compressions were treated with ovalization of the LPA stent followed by LMB dilatation resulting in a mild forward movement of the posterior part of the LPA stent where the LMB balloon was inflated. In the fourth patient LPA stent ovalization and repeated LMB dilatation did not improve LMB diameter.

### 3.4. Clinical Symptoms Caused by Airway Compression

Clinical symptoms related to compression of the airway caused by placement of the LPA stent were reported in two patients. In one patient this manifested in recurrent croup attacks after LPA stenting without airway imaging. In a following procedure, where airway imaging was used, ovalization was performed, causing a reduction of clinical symptoms in this patient. The other patient was described above in which LPA/LMB compression was caused by a large DKS and LMB compression worsened after stenting of the LPA. This patient is 18 years old and symptomatic to date with daily use of nebulizers.

## 4. Discussion

This study shows that periprocedural airway imaging can directly influence the interventional approach in Fontan patients with LPA stenosis. Airway imaging led to an adaption of intervention in 67.1% of the procedures. When LMB proximity was determined, the intervention was modified by either performing ovalization of the LPA stent or refraining from LPA stenting or balloon dilatation. This novel ovalization technique, first described by Krings et al., has been shown to allow successful treatment of LPA stenosis while minimizing compression of the LMB [7]. However, future studies are needed to confirm that periprocedural airway imaging improves clinical outcomes and hemodynamics in the long term in these patients.

In 3/13 procedures, 3DRA resulted in a different assessment of airway patency during balloon interrogation compared with bronchoscopy. In these cases, 3DRA led to misleading information caused by scattering and artifacts by the contrast-filled balloon located next to the LMB. This problem may be overcome by using a balloon filled with pure saline instead of saline/contrast [11]. Bronchoscopy should be considered the gold standard for airway patency in these cases, however, only 3DRA can visualize the vessel–airway relationship [8].

Several anatomical and surgical factors, such as aortic arch reconstruction, repair of the main pulmonary artery, hypoplastic left heart syndrome (HLHS), a large diameter of the ascending aorta and the DKS anastomosis, increase the risk of developing LPA stenosis in patients with single ventricle physiology [2,4]. However, little is known about the incidence of bronchial compression in these patients, with varying rates reported in the literature. O’Byrne et al. reported bronchial compression in 12.5% of patients with hypoplastic left heart syndrome (HLHS) undergoing pulmonary artery stenting [5]. Grohmann et al. reported mild to severe bronchial stenosis related to pulmonary artery intervention in 7 out of 10 HLHS patients with branch pulmonary artery stenosis [12]. In a larger cohort with 120 patients with congenital heart disease, Fernandos et al. reported bronchial compression in 5% of patients following intrathoracic stent implantation, including three cases following LPA stenting, where patients with previous aortic arch reconstruction were at increased risk [13]. This is supported by An et al., who found airway compression in 10.3% of patients with previous aortic arch reconstruction on CT imaging [14].

It is likely that the known risk factors for LPA stenosis are also risk factors for development of bronchial compression. Specifically, patients with HLHS and aortic arch reconstruction may be at higher risk for developing bronchial compression [5,13]. The study by Borik et al. showed that patients who underwent DKS surgery are most likely to develop LMB stenosis, further supporting the importance of surgical anatomy in the development of airway obstruction [6].

Airway compression is a serious complication that is difficult to treat. Because Fontan hemodynamics are highly dependent on an optimal ventilation–perfusion relationship, airway compression may have significant consequences in SV patients [3]. In our series 15 patients had airway compression which was mild in 13 and accepted. None of these patients were symptomatic. When significant LMB compression did occur, airway intervention was performed and consisted of LMB dilatation with or without large vessel dilation and/or manual sternal compression to create a posterior impression in the LPA stent. This was only and partially successful in two of seven procedures and cannot be recommended as therapy of choice, emphasizing the importance of prevention. Hopefully in the future bioabsorbable airway stents can be used to treat severely symptomatic patients [15,16,17].

### Limitations

The retrospective nature of this study, the relatively small cohort and the lack of a true control group are important limitations of this study. Extensive comparison between treatment and control groups was not possible. This study was purely descriptive and it was not possible to identify the most effective and efficient way of airway management.

## 5. Conclusions

The left main bronchus is at high risk for compression when LPA stenting is performed in patients with a Fontan circulation. Periprocedural imaging of the airway has the potential to prevent airway compression. Bronchus dilatation offered no successful treatment for airway compression by an LPA stent. Future studies are needed to confirm long-term beneficial effects of airway imaging.

## Figures and Tables

**Table 1:** Characteristics of the study population. PCPC = Partial Cavo Pulmonary Connection, TCPC = Total Cavo Pulmonary Connection.

	Median [Range]
Age (years)	3.8 [0.4–27.3]
Length (cm)	97 [54–180]
Weight (kg)	14.5 [5–96]
Procedures	1 [1–4]
Procedures performed after PCPC	41
Procedures performed after TCPC	68

**Table 2:** Hemodynamics of Fontan circulation. PAP = pulmonary artery pressure, EDp = end-diastolic pressure.

	Median [Range]
PAP (mmHg)	15 [10–30]
Wedge (mmHg)	9 [6–18]
EDp (mmHg)	9 [5–16]
LPA gradient (mmHg)	2 [0–6]

**Table 3:** Type of LPA stent during first heart catheterization.

Type Stent	Total
EV3 MegaLD	38
Cook Formula 535	25
No stent data available	3
Genesis	1

## Data Availability

The original contributions presented in this study are included in the article. Further inquiries can be directed to the corresponding authors.

## Abbreviations

The following abbreviations are used in this manuscript:

SV	Single ventricle
TCPC	Total Cavo Pulmonary Connection
LPA	Left pulmonary artery
LMB.	Left main bronchus
3DRA	3 dimensional rotational angiography
MPR	Multiplanar reconstruction
WKZ	Wilhelmina Children’s Hospital
VCS	Vena cava superior
VCI	Vena cava inferior
RPA	Right pulmonary artery
Edp	End-diastolic pressure
PCPC	Partial Cavo Pulmonary Connection
PAP	pulmonary artery pressure
DKS	Damus-Kaye-Stansel
